# Silk Fiber-Reinforced Hyaluronic Acid-Based Hydrogel for Cartilage Tissue Engineering

**DOI:** 10.3390/ijms22073635

**Published:** 2021-03-31

**Authors:** Jan-Tobias Weitkamp, Michael Wöltje, Bastian Nußpickel, Felix N. Schmidt, Dilbar Aibibu, Andreas Bayer, David Eglin, Angela R. Armiento, Philipp Arnold, Chokri Cherif, Ralph Lucius, Ralf Smeets, Bodo Kurz, Peter Behrendt

**Affiliations:** 1 Department of Anatomy, Christian-Albrechts-University Kiel, 24118 Kiel, Germany; stu128008@mail.uni-kiel.de (B.N.); a.bayer@anat.uni-kiel.de (A.B.); philipp.arnold@fau.de (P.A.); rlucius@anat.uni-kiel.de (R.L.); bkurz@anat.uni-kiel.de (B.K.); 2 Department of Oral and Maxillofacial Surgery, Division of Regenerative Orofacial Medicine, University Medical Center Hamburg-Eppendorf, 20251 Hamburg, Germany; r.smeets@uke.de; 3 Institute of Textile Machinery and High Performance Material Technology, 01069 Dresden, Germany; michael.woeltje@tu-dresden.de (M.W.); dilbar.aibibu@tu-dresden.de (D.A.); chokri.cherif@tu-dresden.de (C.C.); 4 Department of Osteology and Biomechanics, University Medical Center Hamburg-Eppendorf, 22529 Hamburg, Germany; fel.schmidt@uke.de; 5 Mines Saint-Etienne, Univ Lyon, Univ Jean Monnet, INSERM, U 1059 Sainbiose, Centre CIS, F-42023 Saint-Etienne, France; david.eglin@mines-stetienne.fr; 6 AO Research Institute Davos, 7270 Davos Platz, Switzerland; Angela.Armiento@aofoundation.org; 7 Institute of Functional and Clinical Anatomy, Friedrich-Alexander University Erlangen-Nürnberg, 91054 Erlangen, Germany; 8 Department of Oral and Maxillofacial Surgery, University Medical Center Hamburg-Eppendorf, 20251 Ham-burg, Germany; 9 Clinic for Orthopedic and Trauma Surgery, University Medical Center Schleswig-Holstein, Campus Kiel, 24015 Kiel, Germany; peter.behrendt@uksh.de

**Keywords:** cartilage, hyaluronic acid, chondrocytes, silk fibroin, autologous chondrocyte implantation, biomaterials, TGF-β1

## Abstract

A continuing challenge in cartilage tissue engineering for cartilage regeneration is the creation of a suitable synthetic microenvironment for chondrocytes and tissue regeneration. The aim of this study was to develop a highly tunable hybrid scaffold based on a silk fibroin matrix (SM) and a hyaluronic acid (HA) hydrogel. Human articular chondrocytes were embedded in a porous 3-dimensional SM, before infiltration with tyramine modified HA hydrogel. Scaffolds were cultured in chondropermissive medium with and without TGF-β1. Cell viability and cell distribution were assessed using CellTiter-Blue assay and Live/Dead staining. Chondrogenic marker expression was detected using qPCR. Biosynthesis of matrix compounds was analyzed by dimethylmethylene blue assay and immuno-histology. Differences in biomaterial stiffness and stress relaxation were characterized using a one-step unconfined compression test. Cell morphology was investigated by scanning electron microscopy. Hybrid scaffold revealed superior chondro-inductive and biomechanical properties compared to sole SM. The presence of HA and TGF-β1 increased chondrogenic marker gene expression and matrix deposition. Hybrid scaffolds offer cytocompatible and highly tunable properties as cell-carrier systems, as well as favorable biomechanical properties.

## 1. Introduction

Articular cartilage regeneration is still challenging due to its limited intrinsic self-regenerative capacity [1,2]. Autologous chondrocyte implantation (ACI), a tissue engineering approach, holds great potential for successful regeneration and has become the gold standard for the treatment of large-sized focal cartilage lesions [3]. Since its introduction by Mats Brittberg in 1994, ACI has evolved towards techniques that are based on different biomaterials being used as cell-carrier systems [4]. Third-generation ACI products provide a three-dimensional (3D) cell-carrier matrix, which is either composed of a hydrogel made of natural biopolymers such as agarose–alginate, hyaluronic acid (HA), type 1 collagen, or macroporous scaffolds made of natural materials such as type 1/3 collagen, HA, HA/fibrin or synthetic polymers (e.g., polyglycolic acid and poly(lactic-co-glycolic)acid) [5,6,7]. Although each biomaterial can provide individual advantages for cell viability and differentiation, biomaterials composed of a sole biomaterial are often limited in their overall function. In particular, material stiffness and associated resistance to biomechanical forces, osmotic capacity, degradation behavior and matrix retention have been proven to significantly guide cell differentiation [8,9,10].

Hybrid scaffolds consisting of two biomaterials may enhance cartilage-like tissue properties by recreating its hydro-elastic nature: (i) a fiber network surrounding the encapsulated cells similar to lacunas; and (ii) an osmotic phase restricted by this network resulting in fluid inflow. Attractive biomaterial candidates for this purpose are silk matrices (SM) and HA-based hydrogels. Due to its versatility and high cytocompatibility, silk has gained increasing interest, especially in the field of orthopedic surgery aiming to regenerate ligaments, intervertebral discs, as well as bone [11,12,13]. Its advancing processing technologies open the possibility to fabricate individual 3D structures, which makes silk an ideal biomaterial to design cell-carrier systems [14,15]. Hydrogels were originally developed for their minimally invasive applicability. Particularly, gels based on naturally derived biopolymers such as HA are attractive due to their promotion of chondrogenesis and chondroprotective effects [16,17]. HA can be modified with tyramine (HA-Tyr) to then be crosslinked using horseradish peroxidase (HRP) and hydrogen peroxide (H_2_O_2_) [18]. By varying HRP and H_2_O_2_ concentration, the polymeric network and the mechanical properties of the hydrogel can be adjusted. This has recently been introduced for chondral repair, because it allows TGF-β1 activation and encapsulates medicinal signaling cells (MSC) producing sulphated glycosaminoglycans (sGAGs) in response to mechanical loading [19].

Therefore, the objective of this study was to combine two highly tunable biomaterials and investigate their potential additive properties. Crucial aspects such as cell viability, extracellular matrix (ECM) neo-synthesis, and material stiffness were evaluated.

## 2. Results

### 2.1. Viscoelastic Properties of HA-Tyr Vary Depending on Cell Content and Crosslinking Agents

**Figure 1:**
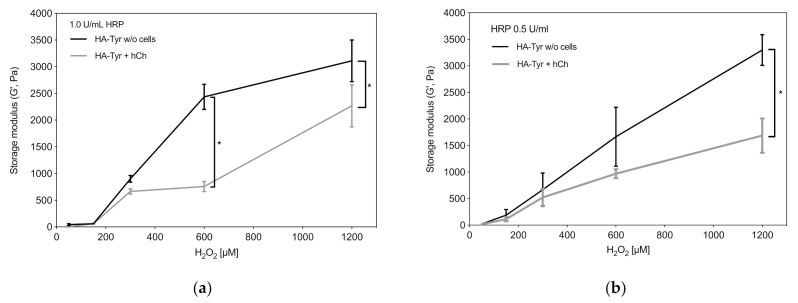
Viscoelastic properties of HA-Tyr depending on cell encapsulation and varying HRP (0.1–1 U/mL) and H_2_O_2_ (50–1200 μM) concentrations. Rheological measurements were performed directly after gelation with (**a**) 1.0 U/mL HRP, and (**b**) 0.5 U/mL HRP, and with and without 2.5 × 10^6^ hCh/mL encapsulated in HA-Tyr. Asterisks indicate significant differences with * *p* < 0.05. Data are presented as the mean ± SD (*n* = 6).

To assess the influence of cell encapsulation on the viscoelastic properties of HA-Tyr hydrogel with different H_2_O_2_ and HRP concentrations, rheological measurements were performed. The results revealed an increase in the storage modulus (G’) with increasing H_2_O_2_ concentrations up to 1200 µM (0.5 U/mL HRP and 600 µM H_2_O_2_ w/o cells: 1665 ± 784.89 Pa; 0.5 U/mL HRP and 1200 µM H_2_O_2_ w/o cells: 3300 ± 410.12 Pa; 1.0 U/mL HRP and 600 µM H_2_O_2_ w/o cells: 2435 ± 332.34 Pa; 1.0 U/mL HRP and 1200 µM H_2_O_2_ w/o cells: 3110 ± 675.35 Pa; Figure 1). Compared to cell-free HA-Tyr, the cell-laden hydrogel had a significantly lower storage modulus (1.0 U/mL HRP and 1200 µM H_2_O_2_ w/o cells: 3110 ± 675.35 Pa; 1.0 U/mL HRP and 1200 µM H_2_O_2_ with cells: 2266.67 ± 680.69 Pa; *p* = 0.0244; Figure 1b). The HRP concentration had a minor influence on the viscoelastic properties, although higher HRP concentration accelerated gelation (data not shown).

### 2.2. Identification of a Cytocompatibility Range of HA-Tyr Crosslinking Agents

**Figure 2:**
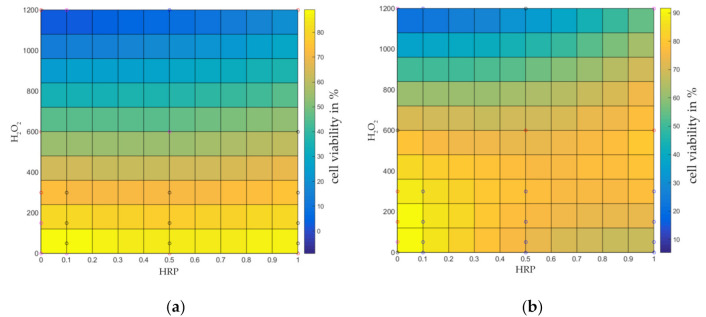
Viability of human chondrocytes depending on varying HRP (0.1–1 U/mL) and H_2_O_2_ (50–1200 μM) concentrations in monolayer. Viability was determined by CellTiter-Blue Assay in 2D-monolayer conditions after 1 day (**a**) and 4 days (**b**). The viability in % is given after normalization to the untreated control group (=100%) and adaptation of the data to a 2-polynomial functional equation using MATLAB r2014 (adjusted *R*^2^ ≥ 0.7) (*n* = 4).

To achieve high primary HA-Tyr stiffness, high concentrations of H_2_O_2_ are needed, as rheological measurements revealed. Subsequently, a cytocompatibility range was evaluated, taking into account the impact of varying concentrations of HA-Tyr crosslinking agents HRP (0.1–1 U/mL) and H_2_O_2_ (50–1200 μM) on cell viability, which was investigated using a CellTiter-Blue assay. Increasing the concentration of H_2_O_2_ significantly affected cell viability of hCh in monolayer, but this effect was mitigated by increasing the HRP concentration (Figure 2). In particular, in the range of high H_2_O_2_ concentration (>1000 µM), 1.0 U/mL HRP led to a distinct increase in cell viability (0.5 U/mL HRP and 1000 µM H_2_O_2_: 12 ± 2.53%; 1.0 U/mL HRP and 1000 µM H_2_O_2_: 37 ± 6.32%). In addition, when exposed to H_2_O_2_ > 600 µM, hCh showed a time-dependent recovery of cell activity, as demonstrated by the results at 96 h. This effect was most significant when using 1.0 U/mL HRP (0.5 U/mL HRP and 1000 µM H_2_O_2_: 49 ± 4.25%; 1.0 U/mL HRP and 1000 µM H_2_O_2_: 68 ± 5.78%; Figure 2b). Based on these results as well as rheological data, HA-Tyr for the hybrid scaffold was prepared using 1000 µM H_2_O_2_ and 1.0 U/mL HRP for all the following experiments.

### 2.3. Cell Viability and Morphological Changes in 3D Cultures

**Figure 3:**
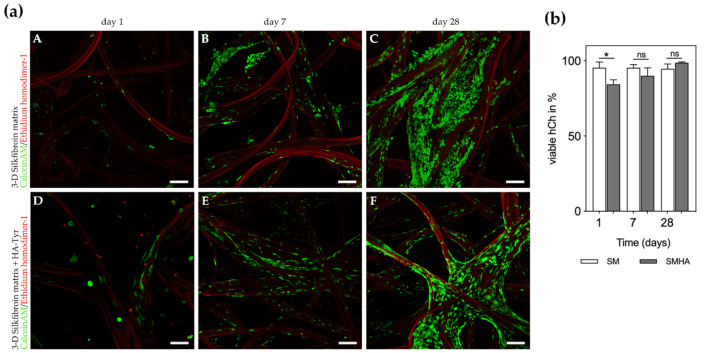
Live/Dead-staining (**a**). **A**–**F**: Viable chondrocytes and indicated proliferation after 1, 7 and 28 days of culture in chondropermissive medium. **A**–**C**: hCh embedded in SM, **D**–**F**: hCh embedded in SMHA. Silk fibers appear red due autofluorescence. Bar 150 µm. Quantification of cell viability (**b**). Viable cell quantification in % of total chondrocytes embedded into SM and SMHA after 1, 7 and 28 days of culture in chondropermissive medium. Asterisks indicate significant differences with * *p* < 0.05, ns = not significant. Data are presented as the mean + SD (*n* = 3).

Human articular chondrocytes cultivated in a silk fibroin matrix (SM) and SM with additional hyaluronic acid-based hydrogel (SMHA) showed high viability (SM: >95% viable cells after 1, 7 and 28 days; SMHA >90% viable cells at day 7 and 28) as quantified from live/dead (L/D) staining (Figure 3). Cell viability in SMHA was initially reduced to 84.3 ± 3.1% (*p* = 0.0245) but recovered within seven days to 90 ± 5.3% (Figure 3b). TGF-β1 had no influence on cell viability (data not shown).

**Figure 4:**
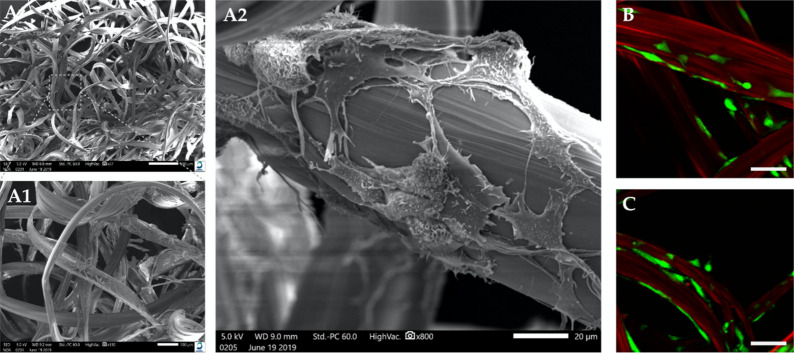
SEM of SM and chondrogenic phenotype. (**A**) Heterogenous and macroporous 3D scaffold architecture. Silk fibroin fiber diameter is approx. 50–100 µm. Chondrocytes revealed a fibroblastic-like phenotype after 3 days of cultivation. L/D imaging of chondrocytes embedded in SM (**B**) and SMHA (**C**). Chondrocytes revealed a ramified phenotype in SM and SMHA groups. Bar 25 µm.

During culture, hCh seemed to attach to the silk fibroin fibers and adopted a ramified cell morphology. No significant differences were detected between SM and SMHA. In SMHA scaffolds, very limited migration into the hydrogel was detected. SEM imaging revealed a fibroblastic-like morphology adopted by the hCh when embedded in SM. Similar appearance of cell morphology was seen in L/D-staining in SMHA groups (Figure 4B,C). By adding HA-Tyr to the silk matrix, the chondrocyte morphology did not significantly change. L/D imaging also indicated cell proliferation during in vitro cultivation in both experimental groups (Figure 3a).

The presence of hyaluronic acid did not have a major influence on cell morphology and proliferation in this experimental design (Figure 4B,C).

### 2.4. Chondrogenic Gene Expression Profile

**Figure 5:**
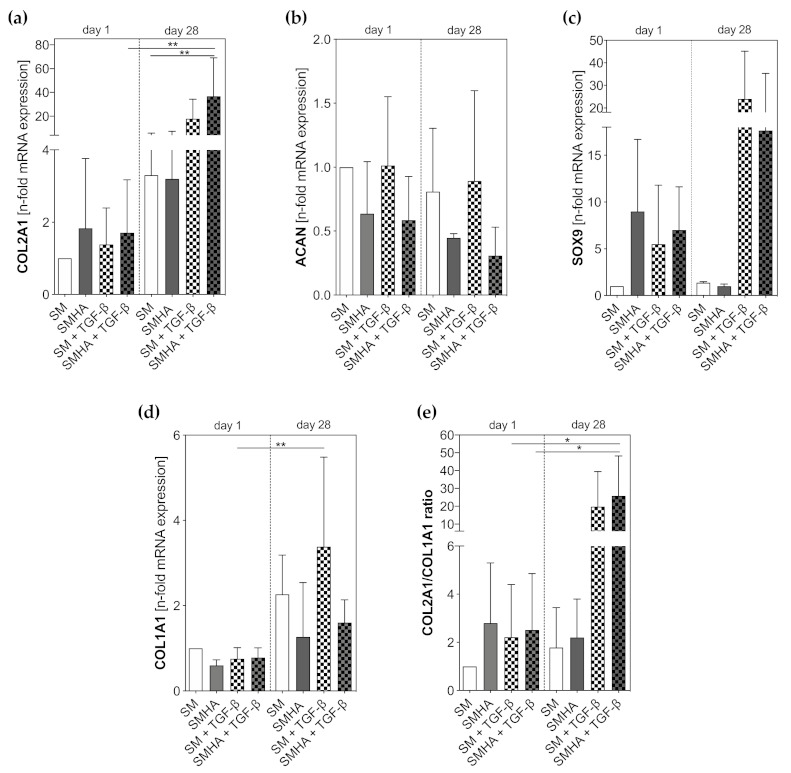
qPCR. Transcription levels (mRNA) of chondrogenic markers: (**a**) COL2A1, (**b**) ACAN, (**c**) SOX9; de-differentiation marker (**d**) COL1A1 and COL2A1/COL1A1 ratio (**e**) were measured at 1 and 28 days of culture in chondropermissive and chondrogenic medium after cell embedding into the biomaterials. Gene expression levels were normalized to those of GAPDH reference gene and then normalized to non-treated SM group, which had an expression level = 1. Asterisks indicate significant differences with * *p* < 0.05, ** *p* < 0.01. Data are presented as the mean + SD (*n* = 3).

Time-dependent increases in CO2A1 and COL2A1/COL1A1 ratios were measured in cells cultured in all different biomaterial combinations. The most pronounced effect was detected in experimental groups treated with TGF-β1 (mRNA expression of COL2A1 in SMHA + TGF-β1 day 1 vs. SMHA + TGF-β1 day 28: *p* = 0.0018; mRNA expression of COL2A1/COL1A1 in SMHA + TGF-β1 day 1 vs. SMHA + TGF-β1 day 28 *p* = 0.0414; Figure 5a,e). During cultivation with TGF-β1, COL2A1 and SOX9 were markedly increased in SM and SMHA groups (Figure 5a,c). No distinct effect of HA on chondrogenic marker gene expression was seen after 28 days. For COL1A1, the presence of HA yielded less COL1A1 expression after 28 days compared to TGF-β1 (Figure 5d). Cells embedded in SM time-dependently induced COL1A1 mRNA expression, which turned out to be significant in the presence of TGF-β1 (*p* = 0.0015).

### 2.5. Chondrogenic ECM Neosynthesis

**Figure 6:**
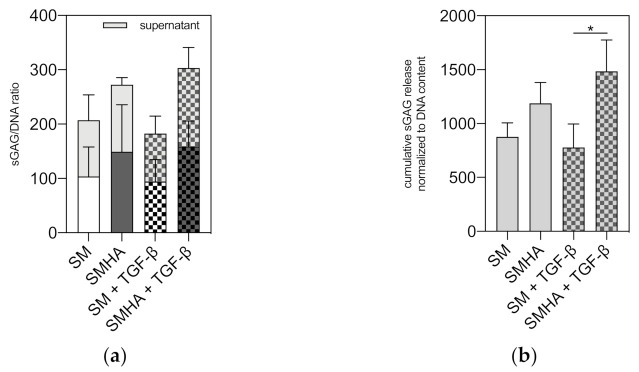
Quantification of sulphated glycosaminoglycan (sGAG) biosynthesis. sGAG content and release normalized to the DNA content in SM and SMHA groups after 28 days of culture in chondropermissive and chondrogenic medium (**a**). Cumulative sGAG release into cell culture supernatant over 28 days of cultivation (**b**). Asterisks indicate significant differences with * *p* < 0.05. Data are presented as the mean + SD (*n* = 3).

Modified 1,9-dimethylmethylene blue (DMMB)-assay indicated higher total glycosaminoglycan biosynthesis normalized to DNA content in SMHA scaffolds compared to sole SM without reaching statistical significance (Figure 6a). Supplementation of TGF-β1 led to no distinct changes of total extracellular matrix production compared to non-treated SM and SMHA (Figure 6a). Cumulative sGAG release over 28 days of culture into the cell culture supernatant revealed similar results. The combination of SM with HA-Tyr hydrogel showed a higher sGAG release over 28 days of cultivation, but also higher sGAG retention within the scaffold. TGF-β showed a minor induction of sGAG release in SMHA groups, while there was no effect on sole SM. Cumulative sGAG release was significantly higher in SMHA + TGF-β groups compared to SM+TGF-β (*p* = 0.0239; Figure 6b).

**Figure 7:**
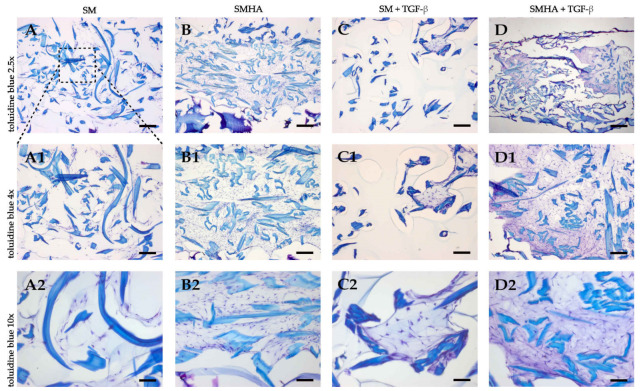
Histological analysis. Toluidine blue staining of sGAGs in cellularized SM (**A**,**C**) and SMHA (**B**,**D**) with or without TGF-β1 supplementation. Representative images of each treatment group after 28 days of culture. Bar 100 μm (**A1**–**D1**, 50 µm; **A2**–**D2**, 25 μm).

Results of sGAG quantification were paralleled by toluidine blue staining. The staining is based on an acidophilic metachromatic dye which selectively stains acidic tissue components such as sGAG, which results in a color change from blue to purple. Samples containing HA-Tyr showed stronger purple staining after four weeks (Figure 7). This effect was enhanced in groups treated with TGF-β (Figure 7(D2)).

**Figure 8:**
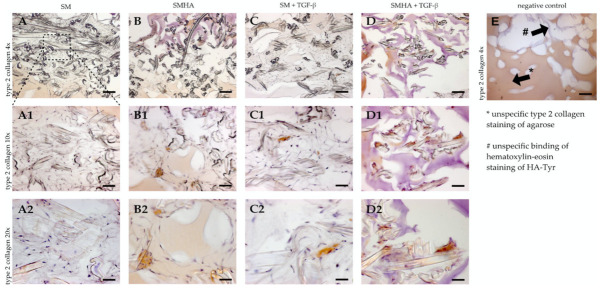
Immunohistochemistry. Type 2 collagen biosynthesis in cellularized SM (**A**,**C**) and SMHA (**B**,**D**) with or without TGF-β1 supplementation. Representative images of immunohistochemical type 2 collagen staining after 28 days of culture. Negative control (**E**) shows unspecific binding of type 2 collagen to agarose and hematoxylin–eosin staining to HA-Tyr. Bar 100 μm (**A1**–**D1**, 50 µm; **A2**–**D2**, 25 μm).

Immunohistochemistry revealed a similar pattern for type 2 collagen staining. SMHA groups showed improved type 2 collagen staining compared to SM with and without TGF-β (Figure 8). HA-Tyr and TGF-β tended to have a synergistic effect on type 2 collagen accumulation, because SMHA+TGF-β samples showed the most pronounced effects (Figure 8(D2)).

### 2.6. Biomechanical Analysis

**Figure 9:**
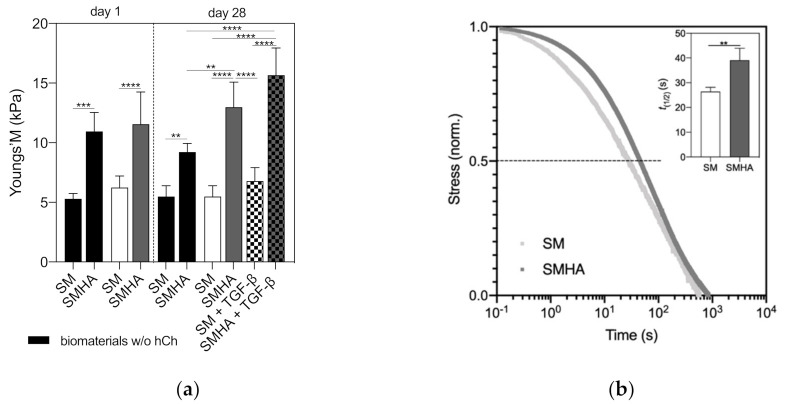
Biomechanical analysis. Young’s modulus (**a**) of SM and SMHA with and without embedded hCh at day 1 and day 28 of culture in chondropermissive and chondrogenic medium (*n* = 6). Stress relaxation time (s) *t*_(1/2)_ of cellularized SM and SMHA after 28 days of culture (**b**). One representative sample is plotted of *n* = 3 measurements. Asterisks indicate significant differences with ** *p* < 0.01, *** *p* < 0.001 and **** *p* < 0.0001. Data are presented as the mean + SD.

A one-step unconfined compression test revealed a significant increase in scaffold stiffness by adding HA-Tyr to the SM (Figure 9a). Primary stiffness of SMHA was significantly higher compared to sole SM (*p* = 0.0003). The presence of hCh did not influence biomaterial stiffness after one day. After 28 days, the Young’s modulus of cell-laden SMHA was significantly higher compared to all SM groups and cell-free SMHA. Treatment with TGF-β1 resulted in an increase in sample stiffness without reaching statistical significance compared to non-treated SMHA groups. Stress relaxation times (t_1/2_) of cell-laden SM and SMHA after 28 days were significantly increased by adding HA-Tyr to SM (SM 26.38 ± 1.77 s vs. SMHA 39.12 ± 4.76 s; *p* = 0.0075; Figure 9b).

## 3. Discussion

In this study, we introduced a hybrid scaffold for cartilage regeneration by utilizing an HA-Tyr hydrogel and SM. Cellularized SM in which HA-Tyr was passively immobilized showed superior chondrogenic potential compared to sole fiber-based scaffolds. The presence of HA resulted in better cartilage-specific ECM retention. This effect was accompanied by increased initial and maturing biomechanical properties, making it predisposed to mechanical loading.

Similarly to cellularized HA-Tyr using MSCs, the presence of chondrocytes impaired the hydrogel viscoelastic properties as assessed by storage modulus changes (G’) [19]. This effect can be counteracted by increasing H_2_O_2_, although H_2_O_2_ causes dose-dependent cell cytotoxicity of articular chondrocytes. Initial cytotoxicity was marginally mitigated by a simultaneous increase in HRP activity, and when using high HRP concentrations of 1 U/mL, further chondroprotective effects could be established over four days. Therefore, 1 U/mL HRP was chosen for further experiments. In order not to affect hydrogel stiffness and to circumvent cytotoxic effects, we decided to primarily embed cells into SM followed by casting the HA secondarily around the cellularized fibers. An increased stiffness could be reached by exploiting the tunability of HA-Tyr. Nevertheless, we detected a significant decrease in cell viability in SMHA hybrid scaffolds during the first week of cultivation, which was most likely due to direct cytotoxic effects of H_2_O_2_ during in situ crosslinking. However, hCh in SMHA recovered after the first week of cultivation and full scaffold colonization was observed in both experimental groups after 28 days. In this regard, Frauchiger et al. also examined SM supporting cell attachment and viability [13].

Another potential advantage of primary cell seeding onto silk fibroin fibers might be immediate cell matrix interactions. Interestingly, there was no cell migration from silk fibers into HA-Tyr, most likely due to fewer extracellular binding motifs. Cell proliferation and migration only occurred along silk fibers, as observed by confocal microscopy. A different degree of binding affinity of hCh may play a decisive role in this. Cell surface interactions with HA through CD44 might be less determining for cell migration, although it is known that focal adhesion factors are modulated by CD44 binding [20]. Cell–fiber interactions hold high potential in guiding cell differentiation, but fiber diameter seems crucial in this regard [21]. In hydrogels, crosslinking density, chemoattractant cofactors, as well as chemical modifications enable cell migration in HA-based hydrogels. However, to the best of the authors’ knowledge, it is not yet known whether early cell migration is beneficial in cartilage defects [22].

In contrast, direct cell–fiber interactions might also be unfavorable in our study. While spherical morphology indicates differentiated chondrocytes, cells attached to SM displayed a rather ramified phenotype. In addition, the presence of HA-Tyr did not alter chondrocyte morphology, although it is known that cells encapsulated in hydrogels adopt a spherical cell shape [23,24]. As highlighted by Mohan et al. and Ziadlou et al., an increasing density of fibers in composite hydrogels influences cell morphology to change from spherical to fibroblast-like [25,26]. On the other hand, Nürnberger et al. postulated that fiber diameters <10 µm in fiber-based scaffolds result in spherical cell shapes [21]. Thus, a fiber diameter of about 100 µm as used in our study may contribute to cell spreading due to a relatively large outer surface. For this reason, thinner fibers might be advantageous in further scaffold optimization.

Although no effect of HA on cell morphology was detected, gene expression patterns and ECM deposition changed significantly. This is particularly important because cell expansion causes chondrocyte dedifferentiation. Re-differentiation and neo-synthesis of ECM is essential for functional graft maturation in vivo. Phenotype loss in monolayers is often characterized by gene expression shifts from COL2A1 towards COL1A1 [27,28]. We observed that TGF-β1 mainly supported COL2A1 induction. Without the presence of the HA, chondrocytes tended to induce COL1A1 expression, which emphasizes the positive effect of hydrogel addition on phenotype stabilization. Furthermore, histology studies revealed that the presence of HA had distinct effects on sGAG and type 2 collagen deposition as well. While the chondro-inductive effects of TGF-β1 are well accepted in the literature, these results support the application of HA in composite scaffolds.

Moreover, besides a support of cellular behavior, HA addition significantly increased the initial scaffold stiffness, which is likely to be due to chemical interconnection of silk fibers by HA-Tyr crosslinking in situ. Silk hydrogels and silk fibroin can be chemically crosslinked with the H_2_O_2_–HRP system through the covalent binding of tyrosine residues, which is similar to the chemical crosslinking reaction of HA-Tyr [29,30]. Deposition of ECM seen in the histology results was paralleled by increased scaffold stiffness in the course of cultivation, while HA and TGF-β1 synergistically enhanced this effect. Mechanically resilient HA-Tyr has been demonstrated to promote differentiation of MSCs towards chondrocyte-like cells by multiaxial mechanical loading through endogenous TGF-β1 activation [19]. Therefore, initial mechanical scaffold strength is highly desirable in order to allow mechano-induced chondrogenic maturation. This is particularly important because the most distinct chondrogenic effect on cell differentiation was observed by TGF-β1 supplementation. Together with further fiber diameter modifications as mentioned above, covalently bonded growth factors could be a potential solution to further improve cell and graft maturation. In addition to scaffold stiffness, a stress relaxation time <63 s for SM and SMHA can be interpreted as favorable biomechanical properties, which has recently been introduced as a meaningful parameter for chondrogenesis [31].

## 4. Materials and Methods

### 4.1. Articular Chondrocyte Isolation and Culture

Human chondrocytes (hCh) (*n* = 6) were isolated from the femoral heads of patients (age 63 ± 5.8 years old) undergoing hip replacement surgery (Ethic votum was obtained from local committee of the University Kiel, Code: D558/19, Date: 5 December 2019). The cartilage tissue was dissected into small pieces and washed twice with phosphate buffer saline (PBS; Sigma-Aldrich, Buchs, Switzerland) for 15 min. Cartilage pieces were first digested with 0.1% pronase (Roche, Mannheim, Germany) for 2 h and then with type 2 collagenase (Worthington, Lakewood, NJ, USA) with an enzyme activity of 600 U/mL for 14 h. Digestion solutions were prepared in Dulbecco’s modified Eagle’s medium (DMEM; Biochrom, Berlin, Germany) and the digestions were carried out at 37 °C in an atmosphere of 5% CO_2_. The digested tissue was filtered through a 40 μm cell strainer and then centrifuged at 565× *g* for 7 min. The obtained cell pellet was washed twice in DMEM supplemented with 10% Sera Plus (PAN-Biotech, Aidenbach, Germany). Isolated chondrocytes were cultured at a density of 10,000 cell/cm^2^ in high-glucose DMEM (HG-DMEM) supplemented with 10% Sera Plus, 10,000 units/mL Penicillin G, 10 mg/mL of streptomycin (PAA Laboratories, Pasching, Germany), 0.1 mM nonessential amino acids (Sigma-Aldrich, St. Louis, MO, USA), 1% L- ascorbic acid (Sigma-Aldrich, St. Louis, MO, USA) and 2 ng/mL fibroblastic growth factor-2 (FGF-2; R&D Systems, Minneapolis, MN, USA), with medium (25 mL/T175 flask) changed twice per week. Cells were harvested at passage 2 by trypsin-EDTA (Lonza; Cologne, Germany) treatment and used for biomaterial colonization.

### 4.2. Embedding of Cells in 3-Dimensional Silk Matrices

Monolayer expanded chondrocytes (cell passage 2) were seeded into a 3D SM (diameter: 6 mm, height: 3 mm) with a final concentration of 2.5 × 10^6^ cells/cm^2^ and incubated for 45 min. The source of silk fibroin was larvae of the silkworm *Bombyx mori*. Larvae were dissected and native silk was extracted from silk glands, as described by Rheinnecker et al. [32]. Then, native silk fibroin solution was utilized to spin fibers directly. Finally, 1.5 g silk short fibers for 200 cm^2^ non-woven fibers were needle-punched, and the resulting non-woven scaffold was subsequently folded in the middle and punched again. For cell culture assays, the needle-punched non-woven scaffolds were then cut into the required diameters. Half of the cellularized scaffolds were then soaked in HA hydrogel for 5 min. HA-based hydrogel (280 kDa) and 6% tyramin substitution (molar degree of substitution) were provided by AO Research Institute Davos and used for all the experiments. For hydrogel preparation, 3.5% (weight/volume) HA-Tyr was hydrated in PBS containing 1.0 U/mL HRP overnight at 4 °C under agitation. Hydrogel gelation was initiated by adding H_2_O_2_ with a final concentration of 0.6 mM [19]. SM and SMHA scaffolds were cultivated in chondropermissive medium in a 24-well plate coated with 2% agarose (Sigma-Aldrich, St. Louis, MO, USA). Medium consisted of HG-DMEM supplemented with 1% ITS (Sigma-Aldrich, St. Louis, MO, USA), 0.1 mM nonessential amino acids (Sigma-Aldrich, St. Louis, MO, USA), 1% L-ascorbic acid, 10,000 units/mL Penicillin G, 10 mg/mL of streptomycin and was renewed every 3 days. The two different scaffolds (SM and SMHA) were cultured in sole chondropermissive medium or chondrogenic medium, which contained additional human TGF-β1 [10 ng/mL] (R&D Systems, Minneapolis, MN, USA).

### 4.3. Viscoelastic Properties in Dependency on Cell Encapsulation and Varying H_2_O_2_ and HRP Concentrations

Rheological measurements were performed using an Anton Paar MCR 302 rheometer equipped with a 25 mm parallel plate measuring system (CP-25-1, Anton Paar, Graz, Austria). All measurements were performed at 20 °C, controlled by a Peltier temperature control unit and with a gap size of 0.2 mm. Oscillatory tests (amplitude and time sweep) were performed for each sample. The storage modulus (G’) was measured at a strain of 1%, which was determined to be within the linear viscoelastic region. To examine the influence of cells on the visco-elastic properties of the HA-Tyr, cell-free and cell-laden hydrogels (*n* = 6; each running with 3 technical replicates) with different H_2_O_2_ (50–1200 µmol) and HRP (0.5, 1 U/mL) concentrations were prepared as described above. Each sample was then prepared using a total volume of 0.5 mL. For the preparation of cell-laden hydrogels, hCh was harvested using trypsin-EDTA as described above. A final concentration of 2.5 × 10^6^ cells/mL were resuspended in 50 µL PBS which was subtracted from the hydrated HA-Tyr solution. After overnight agitation, the cell suspension was spirally injected in the hydrated HA-Tyr solution to ensure the homogenous distribution of cells. Hydrogel gelation of cell-free and cell-laden gels was then initiated by adding varying H_2_O_2_ concentrations (50–1200 µmol). Each sample was prepared directly on the measuring plate of the rheometer. To ensure the homogenous distribution of crosslinking agents, samples were briefly vortexed and then measured.

### 4.4. Cytocompatibility Range of HA-Tyr and Cell Viability

A cytocompatibility range of HA-Tyr under varying H_2_O_2_ (50–1200 µmol) and HRP (0.1–1 U/mL) concentrations was identified with monolayer chondrocytes using a CellTiter-Blue assay. After 24 h and 96 h of incubation in chondropermissive medium, a CellTiter-Blue assay was performed according to the manufacturer’s instructions. After 1 h incubation in an atmosphere of 5% CO_2_ and 37 °C, fluorescence was measured using a TECAN plate reader (Tecan, Männedorf, Switzerland).

To visualize the cell viability, live/dead-staining was performed after 1, 7 and 28 days of culture in chondropermissive medium. SM and SMHA were washed with PBS and stained with 10 μM calcein (Sigma-Aldrich, St. Louis, MO, USA) and 5 μM ethidium homodimer-1 (Sigma-Aldrich, Buchs, Switzerland). After 1 h of incubation at 37 °C in a humidified atmosphere of 5% CO_2_, samples were imaged using laser scanning microscopy (LSM 510, Carl Zeiss, Germany). To quantify the number of live and dead cells, three images were taken from three different fields of view and a minimum of 100 cells were counted using image J software (Wayne Rasband, NIH, USA).

### 4.5. Scaffold Architecture and Cell Morphology

For scanning electron microscopy, after 3 days of culture-cell-free and cell-laden SM, samples were fixed overnight at 4 °C in 3% glutaraldehyde (Sigma-Aldrich, Darmstadt, Germany)/PBS, additionally fixed for 60 min with 2% osmium at room temperature (O_S_O_4_; Pasel und Lorei GmbH, Frankfurt, Germany), and subsequently dehydrated in ethanol. The samples were dried via critical point drying (Balzers, Critical point dryer 030, Schalksmühle, Germany), mounted, sputter-coated with gold (Ion Tech LTD, Teddington, UK), and digitally recorded in a top-down view of the surface using a JSM-IT 200 (JEOL GmbH, Freising, Germany).

### 4.6. Gene Expression Analyses by Quantitative Real-Time qPCR

Gene expression was analyzed after 1 day and 28 days of culture. Samples were snap-frozen in liquid nitrogen and pulverized. Afterwards cells, were lysed by the addition of 300 μL RLT-buffer (Qiagen, Hilden, Germany) and 1% 2-mercaptoethanol (Qiagen, Hilden, Germany). Cell lysate was then separated from pulverized scaffold debris by centrifugation using a Qiashredder (Qiagen, Hilden, Germany) column. Total RNA was extracted using the RNeasy mini kit with additional DNase I digestion according to the manufacturer’s instructions (Qiagen, Hilden, Germany). Complementary DNA (cDNA) was obtained by reverse transcription (RT) using Revert Aid H Minus Reverse Transcriptase (Thermofisher Scientific, Waltham, MA, USA). Quantitative real-time PCR (qPCR) was performed using a QuantiTect SYBR^®^ Green RT-PCR Kit (Qiagen, Hilden, Germany) according to the manufacturer’s instructions with a 7500 Fast Real-Time PCR System (Applied Biosystems, Darmstadt, Germany). Human primers (Table 1) for aggrecan (ACAN), type 2 collagen (COL2A1), transcription factor SOX-9 (SOX9), type 1 collagen (COL1A1) and glyceraldehyde 3-phosphate dehydrogenase (GAPDH) (all from Biomers, Ulm, Germany) were used at a concentration of 0.3 μM. Data analysis was performed using a comparative quantification (ΔΔCT-method). With this method, the n-fold RNA expression for the gene of interest was calculated using GAPDH as reference gene and the SM group as calibrator.

### 4.7. DNA Quantification and Glycosaminoglycan Synthesis

Cell culture supernatant of every medium change was collected and stored frozen (−20 °C) until use. After 28 days in culture, SM and SMHA samples were collected and snap-frozen in liquid nitrogen following pulverization. Afterwards, 1 mL deionized water was added and incubated for 30 min at room temperature. Sulphated glycosaminoglycan (sGAG) content in the SM, SMHA and the culture supernatant was detected with DMMB assay (Sigma-Aldrich, Darmstadt, Germany) as previously described [33]. The DNA content of the samples was quantified using the DNA Quantification Kit (Promega, Mannheim, Germany) according to the manufacturer’s protocol. Total sGAG content and cumulative sGAG release was normalized to DNA content of the corresponding sample.

### 4.8. Histological and Immunohistochemical Analysis

After 28 days in culture, samples were fixed for 10 min in 4% paraformaldehyde in PBS at room temperature, embedded in 2% agarose first and then processed for embedding in Paraplast (Sigma-Aldrich, Darmstadt, Germany). Serial sections of 50 μm were cut sagittally through the entire thickness of the samples (top-to-bottom) and then placed on glass slides followed by toluidine blue staining. In addition, immunohistochemistry of type 2 collagen (mouse anti-type-II-collagen antibody; CIIC1, DSHB, Iowa, USA) was performed as described elsewhere [34,35].

### 4.9. Biomaterial Stiffness and Stress Relaxation

Biomechanical 1-step unconfined compression tests were carried out in a standard material-testing machine (Zwick/Roell BZ2.5/TN1S, Ulm, Germany) equipped with a 10 N load cell. The initial sample height (h_0_) was measured after 24 h equilibration with a caliber, and samples were placed in a cell culture dish filled with HG-DMEM. A compression test was performed by loading the sample with a flat-ended indenter (0.02 N preload) at a strain rate of 0.5 mm/min until 30% h_0_ strain was reached. The Young’s modulus was then calculated at the initial linear part of the stress–strain-curve (*n* = 6). To measure the stress relaxation of SM and SMHA, a constant strain of 20% was applied to each sample, while measuring stress over time (*n* = 3). The stress relaxation time (*t*_1/2_) was quantified as the length of time for which the initial stress of the gel was relaxed to half of its original value.

### 4.10. Statistics

All data were tested for normality using the Kolmogorov–Smirnov test. Statistical analysis was performed using Graph Pad prism 5 program (Graph Pad Software Inc., San Diego, CA, USA). One-way ANOVA analysis with Bonferroni’s multiple comparison was used to compare means among the independent experimental groups. Differences were considered significant if *p* ≤ 0.05. Quantitative data in the text are presented as the mean and standard deviation (SD).

## 5. Conclusions

In this study, we introduced a highly tunable hybrid scaffold with chondro-inductive properties that enabled the encapsulation of long-term viable human chondrocytes. The presence of HA improved cartilage-like marker expression as well as initial mechanical strength, making it accessible for biomechanical loading. Future research should deepen the knowledge in cell matrix interactions, taking smaller fiber diameters into account. A multiphasic approach to mimic cartilage fiber architecture and the possible influence of a superficial zone to shear forces might be an interesting follow-up investigation.

## Figures and Tables

**Table 1:** Primer sequences.

Human Target	Sequence (5′–3′) Sense	Sequence (5′–3′) Antisense
ACAN	GAGGCCAGCAGAGAAGATTCTG	GACGCCTCGCCTTCTTGAA
COL2A1	CAACACTGCCAACGTCCAGAT	CTGCTTCGTCCAGATAGGCAAT
SOX9	CTCGGAGACTTCTGAACGAGAG	CGTTCTTCACCGACTTCCTCC
COL1A1	AATTCCAAGGCCAAGAAGCATG	GGTAGCCATTTCCTTGGTGGTT
GAPDH	GCCTCAAGATCATCAGCAATGC	TGGTCATGAGTCCTTCCACGAT

## Data Availability

Data can be requested from the corresponding author.
